# Incretin effect determines glucose trajectory and insulin sensitivity in youths with obesity

**DOI:** 10.1172/jci.insight.165709

**Published:** 2023-11-22

**Authors:** Alfonso Galderisi, Domenico Tricò, Jessica Lat, Stephanie Samuels, Ram Weiss, Michelle Van Name, Bridget Pierpont, Nicola Santoro, Sonia Caprio

**Affiliations:** 1 Yale University, Department of Pediatrics, New Haven, Connecticut, USA.; 2 Department of Clinical and Experimental Medicine, University of Pisa, Pisa, Italy.; 3 Department of Pediatrics, Ruth Rappaport Childrens’ Hospital, Rambam Medical Center, Haifa, Israel.; 4 Department of Medicine and Health Sciences University of Molise, Campobasso, Italy.

**Keywords:** Endocrinology, Metabolism, Diabetes, Glucose metabolism, Obesity

## Abstract

In youths with obesity, the gut hormone potentiation of insulin secretion — the incretin effect — is blunted. We explored the longitudinal impact of the incretin effect during pubertal transition on β cell function and insulin sensitivity. Youths with obesity and 2-hour glucose level ≥ 120 mg/dL underwent a 3-hour oral glucose-tolerance test (OGTT) and an isoglycemic i.v. glucose infusion to quantify the incretin effect. After 2 years, 30 of 39 participants had a repeated OGTT and were stratified into 3 tertiles according to the baseline incretin effect. The high–incretin effect group demonstrated a longitudinal increase in β cell function (disposition index, minimal model [DI_MM_]), with greater insulin sensitivity at follow-up and stable insulin secretion (φ_total_). A lower incretin effect at baseline was associated with higher 1-hour and 2-hour glucose level at follow-up. The high–incretin effect group displayed a greater increase of GLP-1_7–36_ than the moderate- and low-incretin group at baseline, while such a difference did not persist after 2 years. Glucagon suppression was reduced at follow-up in those with low-baseline incretin in respect to the high-incretin group. The incretin effect during pubertal transition affected the longitudinal trajectory of β cell function and weight in youths with obesity.

## Graphical abstract

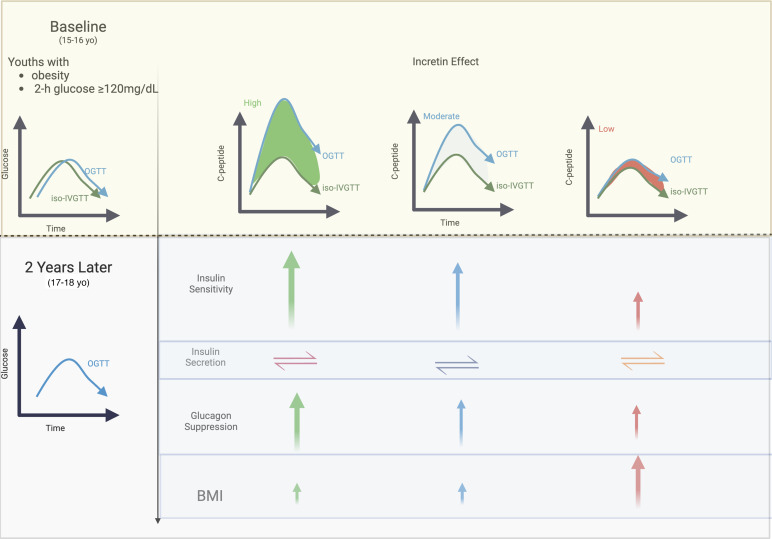

## Introduction

The gut produced incretins — glucagon-like peptide-1 (GLP-1) and gastric inhibitory polypeptide (GIP) — are responsible for an almost ~50% increase of insulin response to orally administered glucose in healthy adults (1), accounting for a large portion of the different insulin response between oral and i.v. glucose load (1). This differential response to oral and i.v. glucose load — namely the incretin effect — declines with progression of hyperglycemia to overt diabetes in both youths and adults (2–5). The temporal sequence of changes of hyperglycemia and incretin effect is still debated with conflicting results (6, 7).

We recently demonstrated that youths with obesity and low-incretin effect exhibit reduced β cell function as compared with their peers with preserved incretin function, in the absence of diabetes (8); however, the long-term effect of incretin response on the trajectory of β cell function and insulin sensitivity (SI) during pubertal transition is still unknown.

More than one-third of U.S. adolescents with obesity have a prediabetes glycemic profile (9, 10), with ~35% progressing to persistent dysglycemia or overt diabetes during adulthood (11). Unlike the gradual prolonged metabolic changes preceding adult-onset type 2 diabetes (T2D), a rapid progression of dysglycemia and β cell failure in the context of insulin resistance occurs in youth-onset T2D (12–15) with at least 1 diabetes complication occurring before the age of 30 years (16). Neverthless, there are no approved drugs to treat prediabetes in youths with a temporally limited window of intervention to prevent diabetes complications after the disease onset.

The identification of the metabolic phenotype of youths with obesity and prediabetes and its natural history with respect to β cell function, SI, and the incretin effect is of pivotal relevance to customize diabetes-preventive strategies targeting the metabolic determinants of youth-onset diabetes to prevent this disease.

Herein, we explore the longitudinal effect of the incretin effect on β cell function in a contemporary cohort of adolescents with obesity and prediabetes evaluated during pubertal transition and 2 years later.

## Results

### Participant characteristics.

Thirty out of 39 participants with a baseline assessment of the incretin effect completed the follow-up oral glucose-tolerance test (OGTT) after 2.2 ± 0.4 years (12 of 13 from the high-incretin, 10 of 13 from the moderate-incretin, and 8 of 13 from the low-incretin groups). Participants had mean ages at baseline of 16.3 ± 2.2 years, 16 were assigned female at birth, and they identified as White (*n* = 5), Black (*n* = 11), and Hispanic (*n* = 14). Participants’ characteristics are displayed in Table 1 according to the baseline incretin tertile. The 3 groups did not differ with respect to anthropometric and metabolic characteristics at baseline and exhibited a similar distribution of gender and ethnicity. Subjects who did not return for the follow-up OGTT did not differ from the analyzed cohort with respect to baseline metabolic and anthropometric characteristics (Supplemental Table 1; supplemental material available online with this article; https://doi.org/10.1172/jci.insight.165709DS1).

While all participants (*n* = 30) had a 2-hour glucose level ≥ 120 mg/dL at baseline, 17 of the 30 participants (57%) maintained a 2-hour glucose level ≥ 120 mg/dL at the follow-up OGTT, with 10 of the 17 exhibiting impaired glucose tolerance (IGT) and 1 exhibiting overt diabetes.

The low-incretin group exhibited a higher BMI at follow-up than baseline (*P* < 0.001) (Table 1), while the other 2 groups did not show any statistically significant changes in BMI (*P* = 0.110 and *P* = 0.410 for the high- and moderate-incretin groups, respectively). Participants were regularly followed up at the obesity clinic, and the lifestyle educational interventions did not differ across the 3 groups. None of the participants was on medications affecting SI during the study period.

### Glucose, insulin, and C-peptide excursions over time.

**Figure 1:**
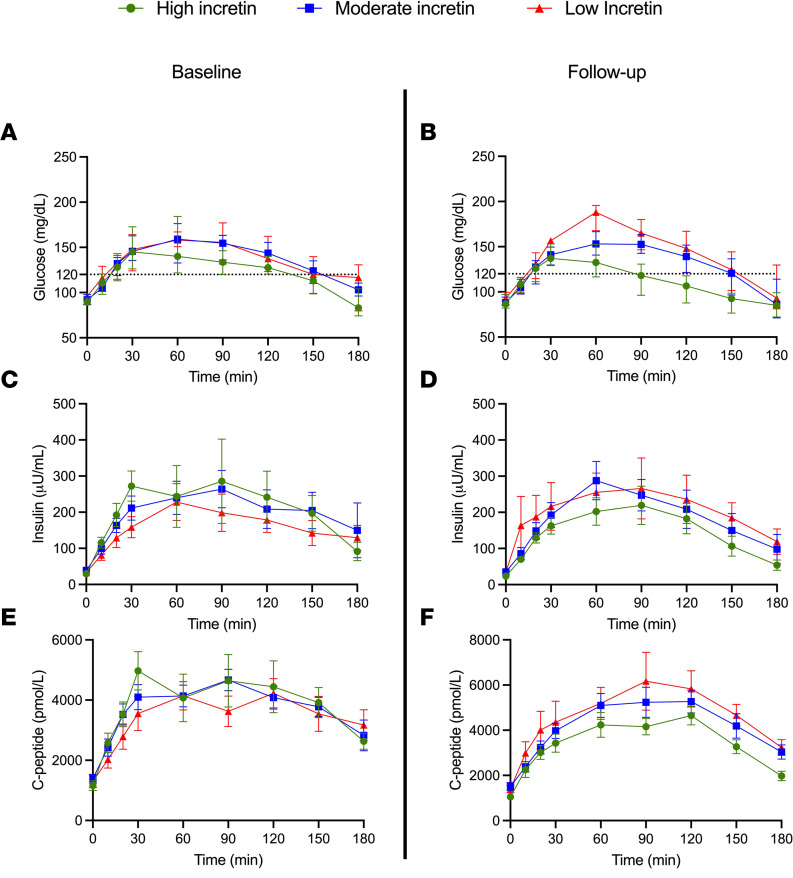
Baseline and follow-up measures of glucose, insulin and C-peptide during the oral glucose tolerance test. Glucose, insulin, and C-peptide profile during the OGTT at baseline (left panels) and at follow-up (right panels) according to the baseline incretin tertile. Data are represented as median and IQR (25th, 75th percentile). Linear mixed model effect analysis has been adopted for comparisons.

At baseline, the 3-hour glucose profile was greater in the low-incretin group (*P* = 0.014) than the high-incretin group, as quantified by the linear mixed model analysis, whereas the glucose response did not significantly differ between the moderate- and high-incretin groups (*P* = 0.098) (Figure 1A). Conversely, insulin excursion was more pronounced in the high- than the low-incretin group (*P* = 0.009), while there was no difference between the moderate- and high-incretin cohorts (*P* = 0.831) (Figure 1C). C-peptide trajectory during the 3-hour OGTT was not different among the groups at baseline (*P* = 0.885 and 0.297 for the high-incretin versus the moderate- and low-incretin cohorts) (Figure 1E).

As displayed in the right panels of Figure 1, at follow up, the greater glucose excursion of the low-incretin group persisted after the oral glucose load (*P* < 0.013) (Figure 1B) in the absence of a significant difference between the moderate- and high-incretin groups (*P* = 0.098). The greater glucose increase over the 3-hour OGTT was paralleled by higher insulin and C-peptide (Figure 1, D and F) concentrations in the low-incretin group compared with the high-incretin cohort (*P* = 0.003 and *P* = 0.031 for C-peptide and insulin).

**Figure 2:**
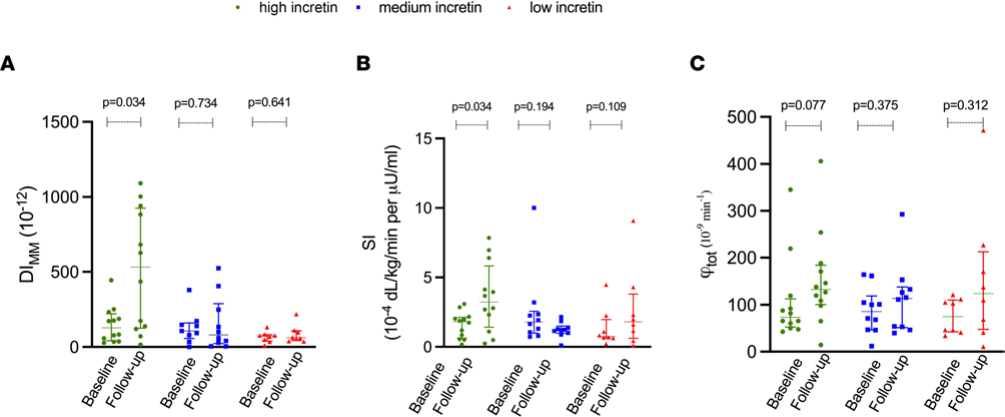
Oral minimal model derived indices of β cell function at baseline and follow-up. (**A**–**C**) Baseline and follow-up β cell function (DI_MM_), insulin sensitivity (SI), and β cell responsiveness (φ_total_) at baseline and follow-up in those with high-, moderate- and low-incretin baseline effect. Data are represented as median and IQR. *P* < 0.05. Kruskal-Wallis test has been adopted for comparisons.

Figure 2 describes the longitudinal changes of β cell function (disposition index, minimal model [DI_MM_]), SI, and β cell responsiveness (φ_total_) in each incretin group as computed through the oral minimal model. Those with high baseline incretin effect demonstrated a > 5-times higher DI_MM_ at follow-up compared with the baseline (*P* = 0.034), while it remained unchanged in the medium- and low-incretin effect groups (*P* = 0.734 and *P* = 0.641) (Figure 2A). This difference was associated with an increase of SI over time (*P* = 0.034) (Figure 2B) and a trend toward a raise of φ_total_ (*P* = 0.077) (Figure 2C) in the high-incretin group.

The cross-group comparison of follow-up β cell function metrics displayed a greater DI_MM_ (Figure 2A) and SI (Figure 2B) in the high-incretin cohort compared with the low-incretin group at follow-up (*P* = 0.044 and *P* = 0.013) in the absence of difference for the β cell responsiveness (φ_total_) (*P* = 0.235) (Figure 2C).

### GLP-1_7–36_ response.

**Figure 3:**
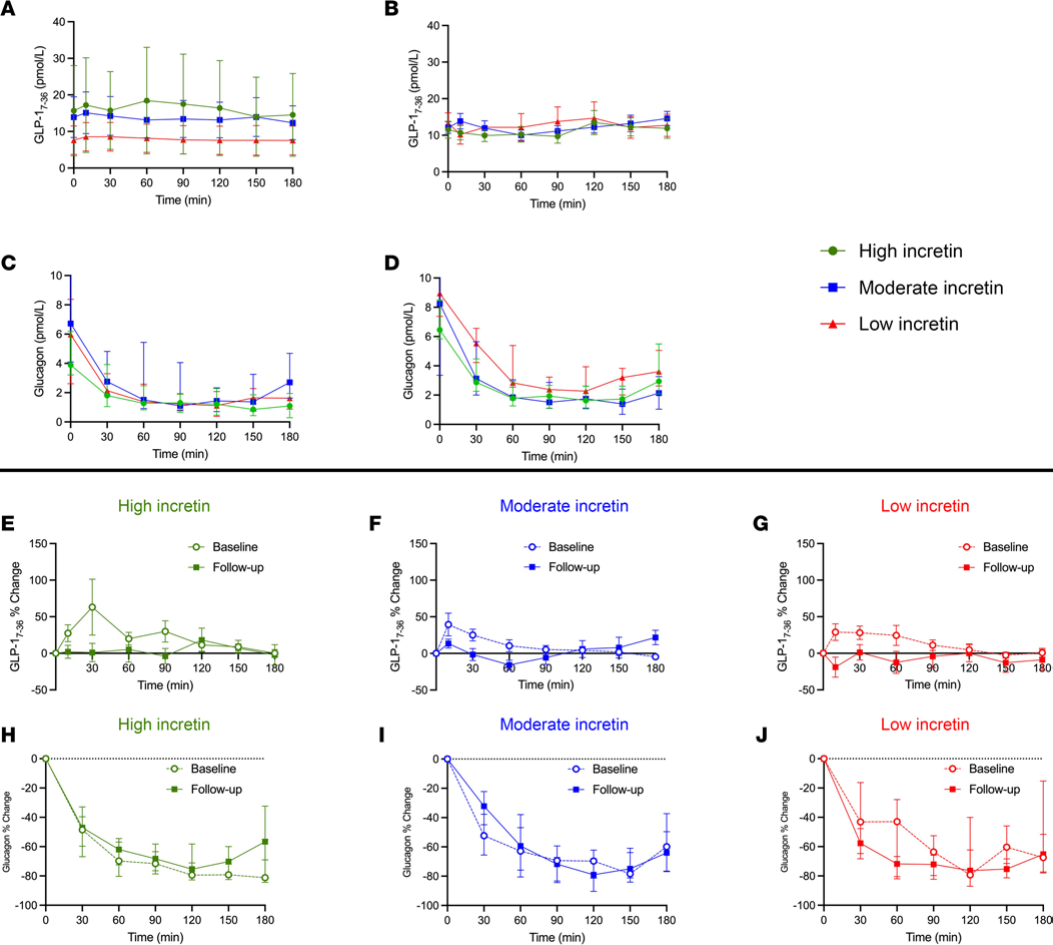
GLP-1 and glucagon response during the OGTT at baseline and follow-up. (**A**–**D**) Baseline and follow-up GLP-1_7–36_ (**A** and **B**) and glucagon (**C** and **D**) for the high-, moderate, and low-incretin groups. (**E**–**G**) Percentage increase from basal for GLP-1_(7–36)_ at baseline and follow-up visits for the high- (**E**), moderate- (**F**), and low-incretin effect (**G**) during the OGTT. (**H**–**J**) Percentage increase from basal for glucagon at baseline and follow-up visits for the high- (**H**), moderate- (**I**), and low-incretin effect (**J**) during the OGTT. Data are represented as median and interquartile ranges (25th, 75th percentile).

At baseline, the high-incretin group displayed a greater fasting GLP-1_7–36_ than the moderate- and low-incretin groups (Figure 3A) (*P* = 0.077 and *P* = 0.048), while this difference was not significant during the follow-up tests (Figure 3B) (*P* = 0.899).

The dynamic GLP-1_7–36_ response during the OGTT at baseline and follow-up is displayed in Figure 3, E–G. The high–incretin effect group displayed a greater percentage increase than the moderate- and low-incretin group during the baseline test (*P* = 0.008 and *P* = 0.029, respectively). Such a difference did not persist at the follow-up OGTT, with blunted excursion across the 3 groups.

### Glucagon response.

At baseline, fasting glucagon did not differ across the 3 groups (*P* = 0.465) (Figure 3C), in spite of a trending higher glucagon of the low and moderate incretin in respect to the high incretin. The high-incretin group showed a lower fasting glucagon at follow-up than those with moderate- and low-incretin effect (*P* = 0.025 and *P* = 0.005, respectively).

While, at baseline, the glucagon excursion during the OGTT did not differ among the 3 groups (Figure 3C), at follow-up, those in the low-incretin group demonstrated a reduced glucagon decrease compared with the high-incretin group (*P* = 0.049) (Figure 3D). This was mirrored by a reduced percentage suppression of glucagon at follow-up in those with low incretin in respect to the baseline suppression (*P* = 0.014) as described in Figure 3J, while we did not observe significant changes in glucagon suppression with respect to the baseline OGTT in those with high- and moderate-incretin effects (*P* = 0.410 and 0.300) (Figure 3, H and I).

### Determinants of glucose tolerance change over time.

**Figure 4:**
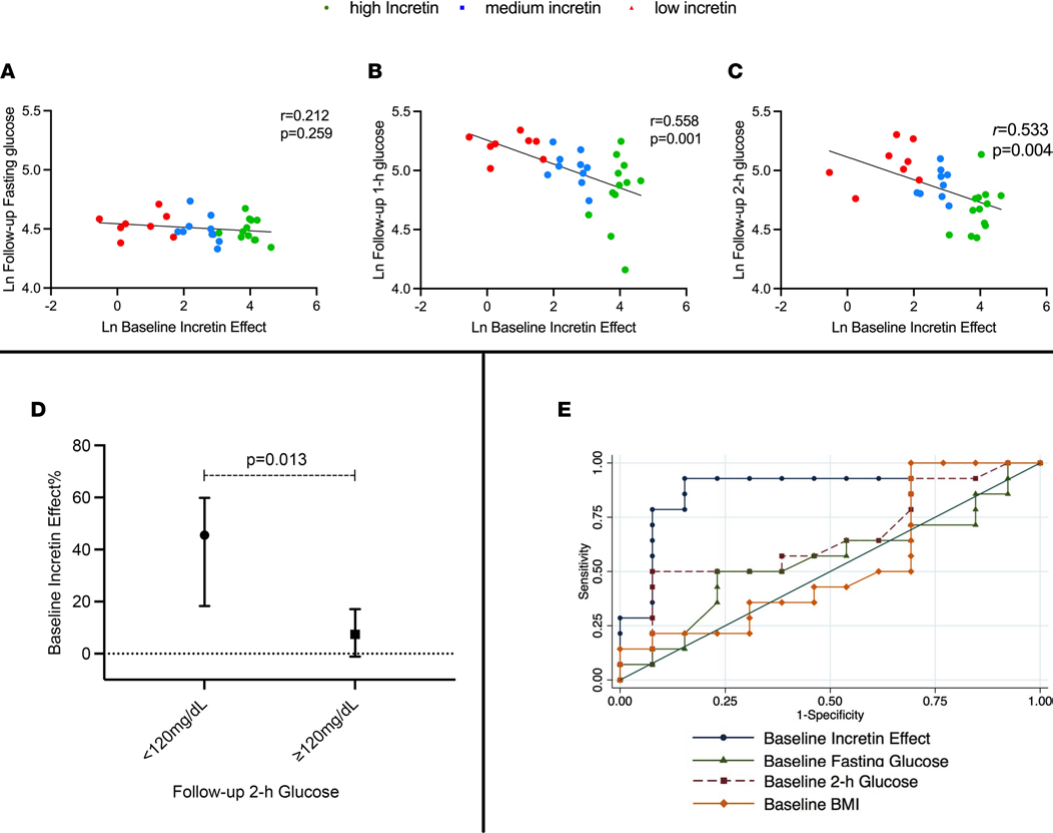
The incretin effect during pubertal transition on longitudinal glucose metrics and BMI. (**A**–**C**) Linear regression analysis of incretin effect and fasting (**A**), 1-hour (**B**), and 2-hour (**C**) glucose. Data are represented as naturally log-transformed measures. Linear regression analysis *r* and *P* value are reported per each variable. (**D**) Baseline incretin effect by 2-hour glucose level at follow-up. Data are expressed as median and IQR (25th, 75th). Kruskal-Wallis test has been adopted for comparison. (**E**) ROC analyses for the binary outcome 2-hour glucose level of ≥ 120 mg/dL at follow-up. The analysis includes baseline incretin effect, baseline fasting, and 2-hour glucose level and baseline BMI as predictors.

In the whole-study cohort, the baseline incretin effect was inversely associated with follow-up 1-hour glucose (*r* = –0.558, *P* = 0.001) and 2-hour glucose level (*r* = –0.533, *P* = 0.004)**,** but not with fasting glucose (Figure 4, A–C). Using a multivariate regression analysis, the baseline incretin effect was a significant determinant of follow-up 2-hour glucose level after adjusting for baseline fasting and 2-hour glucose level, fasting glucagon, BMI, age, and sex (β = –11.0; *P* = 0.035). The baseline incretin effect of those with a 2-hour glucose level ≥ 120 mg/dL at the follow-up OGTT was ~6 times lower than the group returning to 2-hour glucose level (threshold of 120 mg/dL) (45.5% [IQR, 18.3, 59.9] versus 7.4% [IQR, –1.1, 17.0], *P* = 0.013) (Figure 4D), while glucose, C-peptide, insulin, and glucagon profile did not differ between the 2 groups at baseline.

We estimated that a baseline incretin effect equal to or lower than 16.8% would provide 92% sensitivity and 70% specificity to predict a 2-hour glucose level ≥ 120 mg/dL at follow-up in this cohort with an area under the ROC curve of 0.890 ± 0.071. Baseline fasting, 2-hour glucose level, and BMI areas under the ROC curve for the same binary outcome were smaller than the baseline incretin effect (0.552 ± 0.116, 0.648 ± 0.111, and 0.530 ± 0.121, respectively) (Figure 4E).

## Discussion

In this study, for 2 years, we followed-up on 3 groups of youths with obesity stratified according to the incretin effect measured during their pubertal transition by the use of a matched OGTT and isoglycemic venous glucose tolerance test (iso-IVGTT) (1). We demonstrated that a relatively high incretin effect acts as a protective factor toward the β cell function over time. Youths with a low-incretin effect maintain a low–insulin sensitive phenotype after about 2 years, while a high–incretin effect promotes an increase of SI and, as a consequence, an improvement of β cell function. Secondly, we investigated the dynamic changes of the active form of GLP-1 — GLP-1_7–36_ — and glucagon at both baseline and follow-up during the OGTT according to the baseline incretin effect. While GLP-1 excursion was preserved at baseline in those with a high–incretin effect and more pronounced than the other 2 groups, as expected, this difference was not detectable at follow-up (Figure 3, E and F), with flattened GLP-1 excursion in the 3 groups. This suggests that the protective role of incretins during the pubertal transition acts as a priming effect for long-term β cell function, consistently with the higher DI and SI observed in the high–incretin effect group. This observation supports the need for therapeutic interventions preserving the incretin effect during pubertal transition.

Glucagon is physiologically suppressed after the oral glucose load; however, an impaired glucagon suppression has been described in adults with obesity (17). Herein, we observed that a low-incretin effect during pubertal transition is associated with a progressive impairment of glucagon suppression over time. The follow-up measures of β cell function, active GLP-1, and glucagon suggest that the glucagon suppression is primary driven by the preserved β cell function instead of by a contemporary high GLP-1 level.

Our findings are suggestive for the incretin effect to play a priming role during pubertal transition at preserving β cell function and SI over time, and — even in the absence of an adequate response of GLP-1 at later ages — this effect seems to persist and impact both SI and glucagon suppression.

Lastly, the baseline incretin effect demonstrated an inverse linear association with follow-up 1-hour and 2-hour glucose level, supporting the concept of a continuous spectrum of glucose tolerance in youths (18, 19). We adopted a cutoff of 120 mg/dL to explore a potential threshold value for the incretin effect as a predictor of long-term glucose tolerance impairment. This is based on previous reports demonstrating that youths with a 2-hour glucose level equal or greater than 120 mg/dL exhibit a similar levels of both insulin resistance and β cell dysfunction seen in those with 2-hour glucose level ≥ 140 mg/dL (20). Such a threshold has been associated with a 40% reduction of β cell function in youths with obesity with respect to their healthy peers (20, 21). In our cohort, a low baseline incretin effect was predictive for a 2-hour glucose level ≥ 120 mg/dL after ~2 years, regardless the other anthropometric and metabolic baseline characteristics. This observation suggests that a low-incretin effect might be associated with a higher risk for IGT and overt diabetes in adolescents. By adopting the 120 mg/dL cutoff, we identified a clinically significant threshold for the incretin effect in youths with obesity. Lean youths without dysglycemia have been shown to exhibit a ~50% higher insulin secretion during the oral glucose load with respect to the i.v. administration (1, 22, 23); thus, a 50% incretin effect is generally considered as “normal” in the absence of an evidence-based range of values. Obesity with normal glucose tolerance, in turn, is associated with an independent reduction of the incretin effect (~25%) (3), while herein, we describe that a lower threshold — 16% — for the incretin effect would be able to identify > 90% of subjects at risk for a 2-hour glucose level of ≥ 120 mg/dL. This threshold is therefore lower than the average incretin effect previously described in youths with obesity and normal glucose tolerance (3). As displayed by this cohort, the baseline metabolic characteristics of those with a 2-hour glucose level < 120 mg/dL versus ≥ 120 mg/dL did not differ with respect to glucose, insulin, and C-peptide, but those with a persistent 2-hour glucose level ≥ 120 mg/dL exhibited an early defect of incretin effect that, therefore, predates the change in glucose trajectory and the β cell function longitudinal decline.

The main limitation of this study stands in the absence of a longitudinal assessment of incretin effect trajectory by the matched iso-IVGTT. However, the longitudinal measure of the active form of the GLP-1, supports the hypothesis that a priming effect of higher peripubertal higher GLP-1, may provide a persistent metabolic benefit with respect to β cell function and SI. The absence of a measure of other incretins, such as GIP, is an additional limitation of this study. We did not record detailed dietary intakes of the 3 groups over 2 years; however, since they were regularly followed at the same obesity clinic, we do not expect additional confounders and we may infer that the low-incretin effect, per se, might have favored weight gain. The limited numerosity of the groups prevented additional analyses to evaluate the role of other variables — including ethnicity and sex — in the longitudinal progression. Existing evidence from our group and others suggests ethnicity as a major determinant of disease progression and the incretin response in youths with obesity (11, 24).

In conclusion, we demonstrated that a low-incretin effect predates worsening of glucose trajectory over time and is longitudinally associated to a lower SI and higher fasting glucagonemia in youths with obesity.

## Methods

We studied youths with obesity followed at the Yale Pediatric Obesity Clinic and participants enrolled in the “Yale Study of the Pathophysiology of Prediabetes/T2D in Youth” who had body mass index (BMI) > 85th percentile for age and sex, age 8–21 years, and 2-hour glucose level ≥ 120–139 mg/dL.

Exclusion criteria included the use of medications affecting glucose metabolism, a diagnosis of syndromic obesity, or the participation in clinical trials including a structured dietary or exercise-based intervention.

At baseline, participants underwent an OGTT (25) and a matched iso-IVGTT, which reproduced the same plasma glucose profile observed during the OGTT to quantify the incretin effect. The differential insulin secretion between the OGTT and the iso-IVGTT represents the estimated incretin effect with a higher secretion during the OGTT versus the iso-IVGTT being associated with a higher incretin effect.

After 2 years, the OGTT was repeated to evaluate β cell responsiveness, SI, and glucose tolerance status. Tanner stage was determined by a pediatric endocrinologist based on breast development in girls and genitalia development in boys at baseline and follow-up (26, 27).

The baseline characteristics of the original cohort have been described previously (8).

### Procedures and calculations

#### OGTT.

Subjects were admitted to the Yale Center for Clinical Investigation (YCCI) at 8 a.m. after a 12-hour overnight fast. After the local application of a topical anesthetic cream (Emla, Astra Zeneca), 1 antecubital i.v. catheter was inserted for blood sampling. Two baseline samples were then obtained for measurements of plasma glucose, insulin, C-peptide, and glucagon. Thereafter, flavored glucose in a dose of 1.75 g per kilogram of body weight (up to a maximum of 75 g) was given orally, and blood samples were obtained at 10, 20, 30, 60, 90, 120, 150, and 180 minutes for the measurement of plasma glucose, insulin, C-peptide, and glucagon. Glucose samples were immediately processed at the bed side using a YSI2700-STAT-Analyzer (Yellow Springs Instruments).

#### Iso-IVGTT.

Detailed methods of the iso-IVGTT have been reported previously (8). Briefly, participants were admitted within 1 week after the baseline OGTT for an i.v. infusion of dextrose (20%) used to reproduce the plasma glucose profile observed during the OGTT. Frequent adjustments of glucose infusion based on plasma glucose sampling every 5 minutes were adopted to match the profile of the OGTT.

#### Glycemic status.

In accordance with the American Diabetes Association criteria (28), IGT as a 2-hour plasma glucose level between 140 and 199 mg/dL. Pre-IGT status was defined as a 2-hour glucose level of 120–139 mg/dL as previously described (20, 21). Individuals with T2D at baseline were excluded from this study.

#### Biochemical analysis.

Plasma insulin was measured by radioimmunoassay (Linco) that has < 1% cross-reactivity with C-peptide and proinsulin. Plasma C-peptide levels were determined with an assay from Diagnostic Product. Plasma glucagon was measured with the Mercodia Glucagon ELISA (Winston).

The active form of GLP-1 (GLP-1_7–36_) was measured by radioimmunoassay (EMD Millipore) with 0.1% cross-reactivity with GLP-1_7–37_ and GLP-1_1–37_ or other peptides such as human GLP-2, glucagon, human GIP, or vasoactive intestinal peptide.

#### β Cell function in insulin secretion and SI.

Cell function and its components were reported by using both the minimal model estimates. The oral minimal model expresses β cell function (DI_MM_) as the product of the φ_total_ term — based on 9-point measures of plasma glucose and C-peptide during the 3-hour OGTT — and the SI quantified from plasma glucose and insulin during the 3-hour OGTT (29, 30). The computational procedure to estimate the φ_total_ and SI terms has been previously described (8, 29) and was implemented in the SAAM-II 2.3 software (SAAM Institute).

The baseline incretin effect was calculated as the ratio of the difference between the AUC of insulin secretion during the oral (AUC-SR_OGTT_) and the isoglycemic venous (AUC-SR_iso-IVGTT_) glucose tolerance test, over the AUC-SR_OGTT_ and expressed as percentage value (1, 8, 31). A higher value corresponds to a higher insulin secretion during the oral with respect to the i.v. test, thus quantifying a higher incretin effect.

### Statistics

Data were summarized using median (25th percentile, 75th percentile) for continuous variables and count (percentage) for categorical variables.

The original cohort (*n* = 39) was stratified by the baseline incretin effect into 3 tertiles: high- (>66th percentile), moderate- (33rd-66th percentile), and low-incretin (<33rd percentile) effect tertile. The high–incretin effect group was adopted as the comparison term during the analyses (high- versus moderate-incretin and high- versus low-incretin effect group). Paired intragroup analyses were conducted to compare follow-up and baseline values.

Time-series from the OGTT measurements (glucose, C-peptide, insulin, glucagon, and GLP-1_7–36_) were analyzed by linear mixed-model effect. The GLP-1_7–36_ response was additionally evaluated with respect to the baseline value (at time 0) and expressed as a percentage increase from baseline at each time point to normalize the distribution within the cohort.

A linear regression analysis was adopted to test the relationship between baseline incretin effect and fasting, 1-hour, and 2-hour glucose level at follow-up, after adjustment for age, BMI, and sex (32, 33). Tanner stage was collinear with age.

Continuous variables were compared using the Kruskal-Wallis test, followed by post hoc pair-wise Mann-Whitney *U* test. Categorical variables were compared using the χ^2^ test.

ROC curve analysis was also used to seek for the optimal cut point of incretin effect to predict a 2-hour glucose level equal or greater than 120 mg/dL at follow-up. The choice of 120 mg/dL as a threshold was based on available evidence for a decline of β cell function starting above 120 mg/dL in youths with obesity (10, 20).

The analyses have been conducted only for participants who returned for the follow-up assessment.

Analyses were performed using STATA.13 software (StataCorp) and Prism 8.0 (GraphPad Software).

### Study approval

The study protocol was approved by the Human Investigations Committee of the Yale School of Medicine. Participants provided assent, and parents provided written informed consent to participate in the study.

### Data availability

Anonymized data generated during the tests and the codes for the model identification adopted in SAAMII to compute the oral minimal model indices will be available upon request from the corresponding author. Values for all data points in graphs are reported in the Supporting Data Values file.

## Author contributions

AG, SS, JL, and SC performed the metabolic tests. AG ran the model analysis. AG and SC designed the study, collected and analyzed the data, and wrote the manuscript. BP enrolled participants and collected the data. DT and RW contributed to the data analysis and interpretation. NS genotyped the cohort. MVN, RW, NS, and SC critically revised the manuscript. All authors approved the manuscript in its final version. AG and SC are the guarantors of this work and, as such, had full access to all the data in the study and take responsibility for the integrity of the data and the accuracy of the data analysis.

## Supplementary Material

Supplemental data

Supporting data values

## Figures and Tables

**Table 1:**
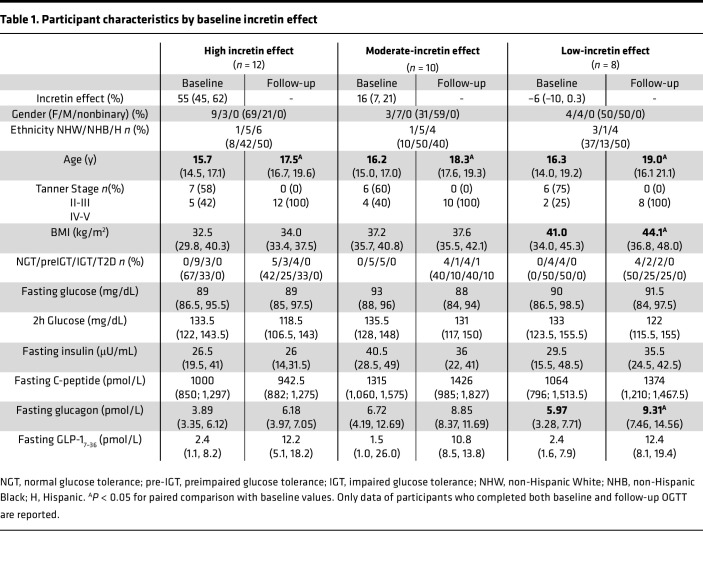
Participant characteristics by baseline incretin effect

## Version 1 10/17/2023

In-Press Preview

## Version 2 11/22/2023

Electronic publication
