# Supplementary material for: Incretin effect determines glucose trajectory and insulin sensitivity in youths with obesity
Source: JCI Insight. 2023 Nov 22;8(22):e165709. doi: 10.1172/jci.insight.165709 (PMC10721315; doi:10.1172/jci.insight.165709)
Supplement: Supplemental data [file jciinsight-8-165709-s160.pdf]

Supplemental Table 1. Baseline characteristics of those who returned for the follow-up OGTT (complete FUP) and those who did not (loos to FUP).

	High Incretin Effect		Moderate Incretin Effect		Low Incretin Effect	
**Baseline**	**Complete FUP**	**Loss to FUP**	**Complete FUP**	**Loss to FUP**	**Complete FUP**	**Loss to FUP**
**N**	**12**	**1**	**10**	**3**	**8**	**5**
**Age (years)**	15.7 (14.5, 17.1)	18 (18, 18)	16.2 (15, 17)	13(12, 14)	16.3 (14.0, 19.2)	15 (12, 16)
**BMI (kg/m^2)**	32.5 (29.8, 40.3)	44.9 (44.9, 44.9)	37.2 (35.7, 40.8)	41.0 (35.3, 42.6)	41 (34.0, 45.3)	39.8 (37, 41.4)
**NGT/preIGT/IGT/T2D n (%)**	0/9/3/0 (0/67/33/0)	0/0/1/0 (0/0/100/0)	0/5/5/0 (0/50/50/0)	0/2/1/0 (0/70/30/0)	0/4/4/0 (0/38/62/0)	0/1/4/0 (0/20/80/2)
**Sex (F/M/Non-binary) (%)**	9/3/0 (69/21/0)	1/0/0 (100/0/0)	3/7/0 (31/69/0)	2/1/0 (67/33)	4/4/0 (50/50/0)	3/2/0 (60/40/0)
**Ethnicity NHW/NHB/H n (%)**	1/5/6 (8/42/50)	0/1/0 (0/100/0)	1/5/4 (10/50/40)	0/1/2 (0/33/67)	3/1/4 (37/13/50)	4/0/1 (80/0/20)

NGT, normal glucose tolerance; pre-IGT, pre-impaired glucose tolerance; *IGT*, impaired glucose tolerance; T2D, type 2 diabetes; *BMI,* body mass index; *NHW,* non-Hispanic White; *NHB,* non-Hispanic Black; *H,* Hispanic; FUP, follow-up.
